# Comparative Study of the Effects of Tai Chi and Square Dance on Immune Function, Physical Health, and Life Satisfaction in Urban Empty-Nest Older Adults

**DOI:** 10.3389/fphys.2021.721758

**Published:** 2021-10-05

**Authors:** ZhongJun Su, JieXiu Zhao

**Affiliations:** ^1^College of Physical Education and Health, Wenzhou University, Wenzhou, China; ^2^China Institute of Sport Science, Beijing, China

**Keywords:** Tai Chi, Square dance, immune function, physical health, life satisfaction, empty nest elderly

## Abstract

**Objective:** To compare the effects of Tai Chi and Square dance on immune function, physical health, and life satisfaction in urban, empty-nest older adults.

**Methods:** This cross-sectional study included 249 older adults (60–69 years) who were categorized into Tai Chi (*n* = 81), Square dance (*n* = 90), and control groups (*n* = 78). We evaluated immunoglobulin G (IgG) and interleukin-2 (IL-2) levels by enzyme-linked immunosorbent assay (ELISA), natural killer (NK) cell cytotoxicity by MTT assay, physical health indices by physical fitness levels, and life satisfaction by Life Satisfaction Index A (LSIA) scores.

**Results:** Immune function, physical health, and life satisfaction in older adults in the Tai Chi and Square dance groups were significantly better than those in the control group (*P* < 0.05). Regarding immune function and physical health, the Tai Chi group exhibited significantly higher levels of IgG (15.41 ± 0.26 g/L vs. 11.99 ± 0.35 g/L, *P* < 0.05), IL-2 (4.60 ± 0.20 ng/mL vs. 4.45 ± 0.21 ng/mL, *P* < 0.05), and NK cell cytotoxicity (0.28 ± 0.02 vs. 0.22 ± 0.02, *P* < 0.05) than the square dance group, significantly lower waist-to-hip ratio (0.87 ± 0.02 vs. 0.89 ± 0.02, *P* < 0.05), resting pulse (78.4 ± 4.6 beats/min vs. 81.0 ± 3.1 beats/min, *P* < 0.05), systolic blood pressure (132.0 ± 5.2 mmHg vs. 136.2 ± 3.2 mmHg, *P* < 0.05), diastolic blood pressure (80.0 ± 2.6 mmHg vs. 83.0 ± 2.7 mmHg, *P* < 0.05), and significantly higher vital capacity (2978.0 ± 263.0 mL vs. 2628.3 ± 262.8 mL, *P* < 0.05) and duration of one-leg standing with eyes closed (16.2 ± 1.9 s vs. 12.0 ± 1.7 s). However, there was no significant difference in LSIA scores between the Tai Chi and Square dance groups (12.05 ± 1.96 vs. 13.07 ± 1.51, *P* > 0.05). Further, there was a significant correlation between LSIA scores and immune function (*r* = 0.50, *P* = 0.00) and physical health (*r* = 0.64, *P* = 0.00).

**Conclusion:** (1) Both Tai Chi and square dance practitioners had better health outcomes, compared with sedentary individuals; (2) Tai Chi practitioners had better physical health and immune function than Square dance practitioners. (3) Tai Chi and Square dance exercises had similar effects on life satisfaction among urban empty-nest older adults.

**Suggestions:** For urban empty-nest older adults who want to have better physical health and immune function, long-term Tai Chi exercise may be a better choice; however, those who are concerned about life satisfaction can choose either Tai Chi or Square dance exercise.

## Introduction

Due to market reform, economic restructuring, the miniaturization of the family structure, and population aging in China, approximately 50% of the older adults in China are currently empty nesters (Zhen, 2016); it is estimated that by 2030, the proportion will reach 90% (Wang G. et al., 2017). Compared with regular older adults, empty nesters constitute a special group of older adults who are prone to suffering from “empty nest syndrome,” which is characterized by a series of psychological disorders, such as feelings of loneliness, emptiness, and depression, which adversely affect their mental health (Guo and Sun, 2018). Mental health not only contributes considerably to life satisfaction but also to immune function. For example, physical and mental health potentially influence life satisfaction in older adults (Pinto et al., 2016; Lombardo et al., 2018), and psychoneuroimmunology studies have indicated that thoughts, emotional patterns, and psychological dynamics are strongly interrelated with immune response (Vasile, 2020). Another research has demonstrated a positive correlation of increased job satisfaction with natural killer (NK) cell number and plasma immunoglobulin G (IgG) concentration (Nakata et al., 2013) as well as a significant relationship between mental resilience, perceived immune function, and health (Van Schrojenstein Lantman et al., 2017).

Hence, life satisfaction is an important psychological factor reflecting the mental health and quality of life of older empty nesters (Zou and Yang, 2017), and it is an abstract and synthetic concept, which involves spiritual, physical, and social factors of individuals in daily life (Holmes and Dickerson, 1987). Since feelings of loneliness, depression, and emptiness, among others, are common in older empty nesters and are associated with adverse health consequences from both mental and immune health perspectives, an intensified focus on introducing more effective intervention strategies targeted at mitigating these feelings, is imperative. It is also important to improve their mental health, immune function, and life satisfaction.

Currently, non-pharmacological strategies, such as exercise, are becoming more popular because of their multifunctional effects and the uncertain efficacy and possible side effects of pharmacological strategies. To date, several kinds of fitness programs, such as Tai Chi and Square dance exercises, have been adopted by the older population in China. Tai Chi exercise is a traditional Chinese physical exercise characterized by meditation and low-to-moderate intensity activity, and it is practiced worldwide by older adults. In addition to improving muscle strength (Manson et al., 2013b; Wehner et al., 2021), balance (Wehner et al., 2021), body mass index (Manson et al., 2013a,b), and systolic blood pressure (SBP) (Manson et al., 2013a), research has also found Tai Chi exercise to have favorable effects on immunity (Yeh et al., 2006; Ho et al., 2013)as well as physical and mental health in older adults (Holly and Helen, 2012; Zheng et al., 2017). Square dance is considered an expansion of line dancing and was introduced in 2004, to China (Li, 2011). Public places where dance sessions are usually conducted consist of music, companions, and leader(s); further, because it is easy to learn and it produces a cheerful atmosphere, Square dance is significantly popular among middle-aged and older Chinese adults, especially among older women. Research has revealed the positive effects of Square dance on depressive symptoms and quality of life-related mental well-being (Wang et al., 2020), physical health and psychological mood (Sun and Wang, 2020), and immunity (Pei et al., 2013) in older adults. To the best of our knowledge, only a few studies have investigated the effectiveness of Square dance, and no study has comparatively evaluated the effects of Tai Chi and Square dance on mental health and immune function in the older population. Research on the differences in effect on mental health and immune function between Tai Chi and Square dance may offer positive guidance to older adults in selecting an appropriate exercise program.

The purpose of this study was to compare the effects of Tai Chi and Square dance exercises on immunity and life satisfaction in empty-nest older adults. We also aimed to evaluate the effects of these exercise on other physical health indicators, including waist-to-hip ratio, SBP, diastolic blood pressure (DBP), vital capacity, resting pulse, and balance. We hypothesized that (1) both Tai Chi and Square dance exercises can have better effect on immunity, physical health, and life satisfaction in empty-nest older adults, (2) Tai Chi exercise has more better effect than Square dance on all the aforementioned indicators in empty-nest older adults, and (3) a significant correlation exists between these indicators.

## Materials and Methods

### Study Population

In this cross-sectional study, 249 empty-nest older adults aged 60–69 years were recruited and categorized into Tai Chi (*n* = 81, female/male [F/M] = 61/20), Square dance (*n* = 90, F/M = 65/25), and control group (*n* = 78, F/M = 60/18). In the Tai Chi and Square dance groups, empty-nest older adults were recruited by cluster sampling and those in the control group with the help of communities. The inclusion criteria for empty-nest older adults in the Tai Chi and Square dance groups were as follows: (1) empty-nest older adults: those without offspring or whose offspring lived in other places; (2) aged 60–69 years; (3) unlimited by gender; (4) engagement in regular exercise for at least 2 years, no less than 120 min/week, and more than 3 times/week; (5) no obvious diseases, such as neurological, cardiovascular, psychiatric, and/or metabolic disease prior to the exercise. The control participants are sedentary due to our choice, because the subjects in Tai Chi or Square dance group only do regular Tai Chi or Square dance exercise, so subjects are sedentary in control group is one of our inclusion criteria.

A sedentary lifestyle was defined as not having participated in exercise for more than once per week for the last year (Audette et al., 2006). All the eligible literate participants provided written informed consent, and for the illiterate ones, the consent statement was read out and signed by the researcher after obtaining their permission. The study’s protocol was approved by Ethics Committee of Wenzhou University (WZU-083).

### Exercise

Tai Chi and Square dance sessions are in the form of a self-organized clubs. Each club has a chief organizer who is responsible for its leadership. Music is being played during exercise. Tai Chi exercises are conducted in the morning (6:00–7:10 a.m.) and Square dance in the evening (7:00–8:10 p.m.), with the exercise venue being a park or square.

### Measures

#### Physical Health

##### Waist-to-Hip Ratio

Waist-to-hip ratio (WHR) is the ratio of the circumference of the waist to that of the hips. We employed measurement methods used by previous researchers (Yang et al., 2017). Waist circumference was measured at a level midway between the lowest rib and the iliac crest using a measuring tape, and hip circumference was measured using the same tape at the widest position of the buttocks, with the tape along a plane parallel to the floor and not compressing the skin, after inhalation and exhalation. Waist and hip circumferences were measured three times for each participant and were accurate to the nearest 0.1 cm, with the average of the three measurements being used for further data analysis.

##### Blood Pressure and Resting Heart Rate

According to the American Heart Association’s standardized protocol (Perloff et al., 1993), we measured SBP, DBP, and resting heart rate (RHR) three times for each participant using an electronic sphygmomanometer (Omron HEM-7071A, Japan), after having them sit for at least 5 min. In cases where there was a difference of more than 5 mmHg or 5 beats/min, the two closest values were adopted (Wang P. et al., 2017). We encouraged participants to avoid alcohol, cigarette smoking, coffee, tea, and excessive exercise for at least 30 min prior to measuring their blood pressure and pulse rate (Wang et al., 2012).

##### Vital Capacity

Vital capacity (VC) is the maximum volume of air exhaled slowly and completely after trying to inhale, that is, VC (mL) = tidal volume + expiratory reserve volume + inspiratory reserve volume (Liu et al., 2017).

We measured VC using previously described methods (Huang et al., 2019). Briefly, VC was measured using a spirometer (Jianmin, GMCS-III type A, Xinheng Oriental Technology Development Co., Ltd, Beijing, China) according to the National Physical Health Test standard guidelines of China as per the following protocol: (1) in a standing position, take 1–2 deep breaths; (2) hold the Venturi handle (the pressure hose is above the Venturi); (3) shift the head slightly backward; (4) attempt to inhale deeply until one can no longer breathe in; and (5) subsequently exhale steadily into the mouthpiece for as long as possible until there is no air left. The maximum value was recorded after three acceptable maneuver attempts. The average of the three measurements was used for further data analysis.

##### One-Leg Standing With Eyes Closed

We used the method described in the National Physical Health Test standard guidelines of China. Briefly, upon the assessor’s command, participants were asked to lift the non-dominant leg off the ground and keep their dominant leg vertical; in this position, participants were asked to stand for as long as possible with the time measured to the nearest to 0.01 s using a stopwatch (JinQue, JD-3B, Shanghai Automation Instrument Co., Ltd.). Before the test measurement was conducted, participants practiced 3–5 trials in the same position as that used in the official measurement. The test was stopped when participants were no longer able to maintain the requirements of the test position.

#### Immune Function

Overnight fasting peripheral venous blood (2 mL) was collected by qualified nurses from all participants at approximately the same time (7:30 a.m.) in a vacuum tube (Cangzhou Yongkang Pharmaceutical Products Co., Ltd., China) for measurement of IgG, Interleukin-2 (IL-2) and NK cell cytotoxicity levels, and forbid exersing, drinking and coffee the night before last.

##### Immunoglobulin G and Interleukin-2

We measured IgG and IL-2 levels using previously reported methods (Meng et al., 2019). The blood samples were centrifuged at 10,000 r/min for 10 min; thereafter, we collected the serum to measure the concentrations of IgG and IL-2 using the commercial enzyme-linked immunosorbent assay (ELISA) kit according to the manufacturer’s protocol. Absorbance was measured using an ELISA reader (Bio-Rad, California, United States).

##### Natural Killer Cell Cytotoxicity

We used peripheral blood mononuclear cells (PBMCs) to assess NK cell cytotoxicity. PBMCs were isolated by density gradient centrifugation using Ficoll-Hypaque (Tianjin Haoyang Biological Manufacture Co., Ltd., China) according to the manufacturer’s operation manual. We performed the proliferation and cytotoxicity assays using freshly isolated PBMCs.

##### Natural Killer Cell Isolation and Purification

PBMCs in the middle cloud layer were extracted using density gradient centrifugation and washed twice using phosphate buffer solution (Wuhan Boster Biological Technology Co., Ltd.); To every 10^7^ cells, 70 μL buffer was added for resuspension, followed by 20 μL CD56 magnetic bead antibody, and subsequently incubated at 2–8°C for 15 min; 1 mL buffer was added for uniform mixing, centrifuged at 300 r/min for 5 min, and subsequently resuspended in 500 μL buffer. The MS separation column was placed in a MiniMACS^TM^ separation magnetic field (Miltenyi Biotec, German), and the column wall was wetted with 500 μL buffer before use; the collecting tube was set in place and the resuspended cells placed on the column, and the column was subsequently washed with 3 × 500 μL buffer. Finally, the separation column was separated from the magnetic field, placed on a new collection tube, and 1 mL buffer was promptly injected to flush down NK cells. NK cells were collected and cultured. A small number of NK cells were labeled with CD56-FITC to verify if the purity exceeded 95%.

##### Natural Killer Cell Culture

The NK cells’ density was adjusted to 2 × 10^5^/mL. Inoculation was performed in 96-well plates in the RPMI 1640 culture system containing 10% inactivated fetal bovine serum and IL-2 (100 U/mL) and cultured in a 5% CO_2_ incubator at 37°C.

##### Natural Killer Cytotoxicity Measurement

NK cells cultured for 48 h were effector cells (E), and K562 cells in the logarithmic growth phase were target cells (T), with E:T = 20:1. Simultaneously, three parallel multiple pores, namely, the target cell pore, effector cell pore, and medium blank control pore, were set up and cultured in a 5% CO_2_ incubator at 37°C for 12 h. CCK-8 (10 μL; Dojindo, Japan) was added to each well and recultured for 4 h. Absorbance (OD value) was determined using a microplate reader at 450 nm wavelength as previously described (Mehla et al., 2010), and the average value was used for further analysis. Cytotoxicity = (1– [OD value of effector pore of target cell—OD value of effector pore]/OD value of target cell) × 100%.

#### Life Satisfaction Index A

This scale includes 20 items, and each item has three options, namely, “agree,” “disagree,” and “uncertain.” The total score was the sum of each item, with a score range of 0–20 points; a higher score indicated a higher life satisfaction (Neugarten Bernice et al., 1961).

### Data Analysis

Descriptive statistics were used to present the demographic characteristics of the total population in the three groups. Shapiro-Wilk and Levene’s tests were used for normal distribution and homogeneity test of variance for the continuous data respectively. Continuous variables are presented as mean ± SD and categorical data was expressed as a percentage. One way ANOVA was used to evaluate the differences in immune function, physical health, and life satisfaction data among the three groups, and the Bonferroni *post hoc* test was used to determine significant differences between groups if there is significant differences overall. The chi-squared test was performed to examine the demographic homogeneity among three groups; canonical correlations tests was conducted to evaluate the correlation between the life satisfaction index A (LSIA) and immunity and physical health, of which LSIA was set as aggregation 1; IgG, NK cytotoxicity, and IL-2 were set as aggregation 2; and physical health was set as aggregation 3. Results were considered statistically significant or very significant if their two-tailed *p*-value was < 0.05 or 0.01, respectively. IBM SPSS Statistics software for Windows (version 25; IBM Corporation, Somers, NY, United States) was used for statistical analysis.

## Results

We had conducted a *post hoc* power analysis according to our sample size for statistics power by G^∗^power 3.0.10. The parameter is set as follows: α is 0.05, the effect size is 0.3, and the total sample size is 249, then the calculated power (1-βerr prob) for *F*-test, *X*^2^-test and Correlation-test is 0.99, 0.96–0.99 and 0.99, respectively, belongs to large effect size.

### Demographic Characteristics

There were 81, 90, and 78 participants in the Tai Chi, Square dance, and control groups, respectively, with average ages of 64.4 ± 2.2, 64.6 ± 2.3, and 64.5 ± 2.3 years, respectively. No significant differences were observed in demographic data across the three group, almost three quarters of the participants in the three groups were women. The majority of the participants in the Tai Chi and Square dance groups reported that they had not participated in other forms of regular exercise, except for occasional walking; the same was reported in the control group. No statistically significant differences in demographic variables were observed across the three groups or between Tai Chi and Square dance group (Table 1).

**TABLE 1:** Demographics of the participants in the three groups (*n* = 249).

Variables	Group			X^2^/F	*P*
	Tai chi (*n* = 81)	Square dance (*n* = 90)	Control (*n* = 78)		
Age (Mean ± SD)	64.4 ± 2.2	64.6 ± 2.3	64.5 ± 2.3	0.16	0.86
**Gender**					
Female	61 (75.3%)	65 (72.2%)	60 (76.9%)	0.51	0.77
Male	20 (24.7%)	25 (27.8%)	18 (23.1%)		
**Marital status**					
Married	49 (60.5%)	61 (67.8%)	46 (59.0%)	1.62	0.44
Single or widowed	32 (39.5%)	29 (22.2%)	32 (41.0%)		
**Education**					
None	17 (21.0%)	15 (16.7%)	16 (20.5%)	1.04	0.98
Elementary	47 (58.0%)	55 (61.1%)	46 (59.0%)		
High school	6 (7.4%)	9 (10.0%)	7 (9.0%)		
Special school and above	11 (13.6%)	11 (12.2%)	9 (11.5%)		
**Frequency of exercise/week**					
3–5 times/week	71 (87.7%)	82 (91.1%)	–	0.54	0.46*
≥ 6 times/week	10 (12.3%)	8 (8.9%)	–		
Duration of exercise/time	47.8 ± 5.1	45.2 ± 3.1		0.21	0.52*
**Total years of exercise**					
2–4 years	49 (60.5%)	58 (64.4%)	–	0.70	0.40*
≥ 5 years	32 (39.5%)	32 (35.6%)	–		

*Indicates the significant difference between Tai Chi and Square dance group.

*SD standard deviation.*

### Immunity of the Participants

Compared with the control group, Tai Chi and Square dance significantly improved IgG, IL-2, and NK cytotoxicity levels (*p* < 0.05). Further, the effects of Tai Chi were significantly superior to those of Square dance (*p* < 0.05; Table 2).

**TABLE 2:** Immunity of the participants in the three groups (*n* = 249).

Variables	Tai chi (*n* = 81)	Square dance (*n* = 90)	Control (*n* = 78)
IgG (g/L)	15.41 ± 0.26^#^*	11.99 ± 0.35*	9.92 ± 0.22
IL-2 (ng/mL)	4.60 ± 0.20^#^*	4.45 ± 0.21*	4.09 ± 0.19
NK cell cytotoxicity	0.28 ± 0.02^#^*	0.22 ± 0.02*	0.20 ± 0.02

^#^ and * indicate significant differences when compared with Square dance and Control groups, respectively. IgG immunoglobulin G; IL-2 interleukin-2; NK natural killer.

### Physical Health of the Participants

Compared with control group, Tai Chi had better effects on all the indicators (*p* < 0.05); Square dance had better effects on all the indicators, except for WHR (*p* < 0.05). Moreover, the effects of Tai Chi were significantly superior to those of Square dance (*p* < 0.05; Table 3).

**TABLE 3:** Physical health of the participants in the three groups (*n* = 249).

Variables	Tai Chi (*n* = 81)	Square dance (*n* = 90)	Control (*n* = 78)
WHR	0.87 ± 0.02^#^*	0.89 ± 0.02	0.89 ± 0.02
SBP (mmHg)	132.0 ± 5.2^#^*	136.3 ± 3.2*	140.0 ± 3.0
DBP (mmHg)	80.0 ± 2.6^#^*	83.0 ± 2.7*	89.4 ± 2.7
Vital capacity (mL)	2978.0 ± 263.0^#^*	2628.3 ± 262.8*	2279.1 ± 240.8
RHR (beat/min)	78.4 ± 4.6^#^*	81.0 ± 3.1*	83.6 ± 3.4
OSEC (s)	16.2 ± 1.9^#^*	12.0 ± 1.7*	7.0 ± 1.7

^#^ and * indicate significant differences when compared with Square dance and Control groups, respectively. WHR waist-to-hip ratio; SBP systolic blood pressure; DBP diastolic blood pressure; RHR resting heart rate; OSEC one-leg standing with eyes closed.

### Life Satisfaction of the Participants

In both the Tai Chi and Square dance groups, the LSIA index was significantly higher than that in the control group (*p* < 0.05), and no significant differences were noted between the Tai Chi and Square dance groups (Table 4).

**TABLE 4:** Life satisfaction of the participants in the three groups (*n* = 249).

Variables	Tai Chi (*n* = 81)	Square dance (*n* = 90)	Control (*n* = 78)
Life satisfaction	12.05 ± 1.96*	13.07 ± 1.51*	9.04 ± 1.54

*Indicates significant difference when compared with the Control group.

### Correlation Between Life Satisfaction Index A, Immunity, and Physical Health

Significant correlation was observed between LSIA, immunity and physical health (*p* = 0.00; Table 5).

**TABLE 5:** Correlation between LSIA, immunity and physical health (*n* = 249).

Canonical correlation	Aggregation 1		
	R	F	P
Aggregation 2	0.50	27.28	0.00
Aggregation 3	0.64	28.59	0.00

*Aggregation 1, Life satisfaction index; Aggregation 2, IgG (g/L) + NK cell cytotoxicity + IL-2 (ng/mL); Aggregation 3, Systolic blood pressure (mmHg) + diastolic blood pressure (mmHg) + resting heart rate (beat/min) + one-leg standing with eyes closed (s) + waist-to-hip ratio + Vital capacity (mL).*

## Discussion

The purpose of this study was to compare the effects of Tai Chi and Square dance exercises on empty-nest older adults by mainly measuring the following three indicators: immune function, physical health, and life satisfaction. To the best of our knowledge, this study is the first to compare the effects of long-term practice of Tai Chi and Square dance on immunity, physical health, and life satisfaction in empty-nest older adults.

Our study demonstrated that Tai Chi had a significantly greater effect on immunity and physical health indicators in older adults (*p* < 0.05) compared with the other two groups; however, no such effect was observed with regard to life satisfaction when compared with Square dance (*p* > 0.05). Further, there were significantly different effects on immunity and physical health indicators as well as life satisfaction (*p* < 0.05) between the Square dance and control groups in older adults except for WHR (*p* > 0.05).

The reason underlying the different effects of the Tai Chi and Square dance exercises may be their unique characteristics. Tai Chi exercise integrates physical, psychosocial, spiritual, and behavioral components to promote mind-body interactions (Wang, 2011). It is a moderate-intensity exercise, as no more than 55% maximal oxygen intake is required (Wang et al., 2004), and it should be practiced in harmony with the Tai Chi philosophy by utilizing and manipulating Qi via Tai Chi exercise (Zheng et al., 2017). Qi is a very important concept in Chinese classical philosophy and medicine. It is not a body organ which can be anatomically identified by its location like the chakras of yoga (Cho et al., 2019). In Tai Chi exercise, it is emphasized that “Qi sinks into Dantian,” “Qi runs all over the body,” and “middle Qi passes through the top,” all of which is important to eliminate diseases and improve human function. Smooth flow of Qi makes the body comfortable, and stagnation makes the body sick. Although a deep understanding of the essence of Qi is still lacking, one can feel the existence of Qi while practicing Tai Chi exercise, as smooth flow of Qi improves fingertip numbness, distension, etc.

Square dance, which integrates Chinese style dancing and music with energetic and similar rhythms (Zhou, 2014), introduced in China around 2004 and considered an expansion of line dancing (Li, 2011), is just a medium-intensity exercise. Research shows that Tai Chi exercise has significantly better effect on cognitive function and emotion in older people than Square dance (Zhang et al., 2014), as well as better effect on enhancing lower extremity strength, balance, and flexibility than brisk walking (Audette et al., 2006). Davidson et al. (2003) directly found that an 8-week clinical training program in mindfulness meditation significantly increases the left-sided anterior activation and immune function, and that activated left-sided anterior of the brain was associated with enhanced NK-cell activity (Kang et al., 1991; Davidson et al., 1999). Irwin et al. (2003) confirmed that Tai Chi potentially increases varicella-zoster virus specific cell-mediated immunity in older adults and potentially improves T-helper cell function (Yeh et al., 2009). Tai Chi exercise, which focuses on producing inner calmness, would have both physical and psychological therapeutic value (Docker, 2006). Therefore, we have reason to believe that the comprehensive nature of Tai Chi exercise rendered it significantly superior to Square dance in improving physical health and immune function.

The mechanism underlying Tai Chi exercise’s ability to significantly increase immune function compared with Square dance may be related to a more pronounced increase in antibody titer, telomerase activity, reverse gene expression, reduced DNA damage, etc. Jacobs et al. (2011) found that meditation training could suppress immune cell aging because meditation can significantly increase immunocyte telomerase activity in normal people. Moreover, Goon et al. (2008) found that practicing Tai Chi exercise for 7 years provided a significantly effective DNA repair mechanism, reduced DNA damage, and increased lymphocyte apoptosis and proliferation in older adults; the upregulated lymphocyte apoptosis and proliferation with Tai Chi exercise may also be beneficial in preventing replicative senescence during aging. Our study also found Tai Chi exercise to be significantly effective in improving WHR (*p* < 0.05), which was in accordance with the results of Siu et al. (2021), who found that Tai Chi exercise can significantly decrease waist circumference. A previous study Minuzzi et al. (2018) has indicated that high levels of aerobic fitness may help prevent the accumulation of senescent T-cells during the natural aging process. Davidson et al. (2003) proved that more antibodies against the influenza-virus vaccine were formed in the meditation training group. Buric et al. (2017) found that mind-body interventions can downregulate the nuclear factor kappa B pathway, which antagonizes the effects of chronic stress on gene expression.

Chronic diseases and objective losses in functionality are considered to be strongly related to low levels of life satisfaction in older adults. Pinto et al. (2016) found self-rated health to be a mediator variable between physical and mental health and life satisfaction in older adults. In our study, some participants claimed that after practicing Tai Chi, they seldom caught a cold, even when the weather changed. The body’s innate immune function declines with aging (Solana et al., 2012). Research has shown life satisfaction to be related to physical and mental health (Puvill et al., 2016; Lombardo et al., 2018), and life satisfaction is an important psychological factor reflecting the mental health and quality of life of empty-nest older adults (Zou and Yang, 2017). Hence, physical health, immune function, and life satisfaction significantly correlated (Table 5). Our results are consistent with those of other researchers; for example, Teixeira Vaz et al. (2019) found a positive relationship between physical activity and life satisfaction in older adults.

Compared to Tai Chi exercise, Square dance is a group activity that is more relaxing, less serious, and does not involve Kung Fu. Xie et al. (2021) found the group camaraderie in Square dance to be positively associated with the subjective well-being of middle-aged and empty-nest older women. Therefore, we consider Tai Chi and Square dance to be instrumental in improving empty-nest older adults’ life satisfaction through different mechanisms, that is, through Tai Chi’s movement characteristics and Square dance’s sense of camaraderie; however, no significant difference was observed.

Our study has several limitations. First, the cross-sectional design of this study is not very rigorous because we did not measure the data before and after the experiment, and this has a certain impact on the accuracy of the experimental results; however, we made efforts to control the accuracy by measuring the physical activities of participants. A more precise study design should be a randomized controlled trial, so this cross-sectional design because of the time and financial constraints is the limitation of our research method. Second, the disadvantage of cluster sampling is that the sampling error caused by the large differences between groups is often greater than that of simple random sampling. Third, there are some differences in exercise frequency, time, duration, and standardization of the samples obtained by cluster sampling. Finally, the members in a self-organized club may be those who have similar personalities and a common language and feel happy, which influences the effect of exercise on life satisfaction.

Therefore, these limitations should be considered when interpreting the results of this study.

## Conclusion and Suggestions

Overall, our study demonstrated that both Tai Chi and Square dance exercises promote better physical health, immune function, and life satisfaction in empty-nest older adults; Tai Chi exercise was significantly superior to Square dance in physical health and immune function. Hence, we recommend Tai Chi as the preferred choice of exercise for empty-nest older adults who desire to have better physical fitness and immune function and either Tai Chi or square dance as an equally suitable choice for those desiring to have better life satisfaction.

## Data Availability Statement

The original contributions presented in the study are included in the article/supplementary material, further inquiries can be directed to the corresponding author/s.

## Ethics Statement

The studies involving human participants were reviewed and approved by Ethics Committee of Wenzhou University (WZU-083). The patients/participants provided their written informed consent to participate in this study.

## Author Contributions

JZ was responsible for the overall experimental design, index selection, financial support, manuscript polishing, and data analysis. ZS was responsible for the experiment implementation, index test, and first draft writing. Both authors contributed to the article and approved the submitted version.

## Conflict of Interest

The authors declare that the research was conducted in the absence of any commercial or financial relationships that could be construed as a potential conflict of interest.

## Publisher’s Note

All claims expressed in this article are solely those of the authors and do not necessarily represent those of their affiliated organizations, or those of the publisher, the editors and the reviewers. Any product that may be evaluated in this article, or claim that may be made by its manufacturer, is not guaranteed or endorsed by the publisher.
